# Supplementary material for: Steady-state measures of visual suppression
Source: PLoS Comput Biol. 2021 Oct 13;17(10):e1009507. doi: 10.1371/journal.pcbi.1009507 (PMC8544832; doi:10.1371/journal.pcbi.1009507)
Supplement: Attachment — Submitted filename: coverletterPLoSCB2.docx [file pcbi.1009507.s001.docx]

Department of Psychology

University of York

Heslington

York, UK

YO10 5DD

24^th^ September 2021

Dear Dr Yang,

Please find attached a revised copy of our manuscript that is now entitled “*Steady-state measures of visual suppression*”, and which we wish to resubmit for consideration at *PLoS Computational Biology.* We received supportive and insightful comments from two reviewers and have updated the manuscript accordingly (including changing the title) as detailed below.

We hope that you now find the work suitable for inclusion in the journal, and look forward to receiving your decision.

Yours sincerely,

Daniel Baker, Greta Vilidaite and Alex Wade

BioRxiv preprint: https://doi.org/10.1101/2021.07.27.453123

Code and data: https://dx.doi.org/10.17605/OSF.IO/E62WU

Reviewer #1: This manuscript has strengths and weaknesses. Overall the strengths outweigh the weaknesses and with appropriate revision, it is likely that this manuscript will make a significant contribution to our understanding of contrast masking.

*We thank the reviewer for their constructive and supportive comments.*

R1.1 Review of previous contrast masking studies
Strength: The manuscript evaluates 16 previous studies that examined how a mask interferes with the visibility of a target and concludes that these studies taken together do not show clear evidence for either contrast gain (where the mask reduces the sensitivity to the target) or response gain (where the mask reduces the maximum response evoked by the target). From their analysis of these studies, which include masks that are superimposed or surround the target, or are presented in the other eye (dichoptic), they conclude that there is no clear evidence for either contrast or response gain.
Weakness: 1. The review seems to combine examples that show different changes to the contrast response function in the presence of a mask (e.g., it appears that the 3.3 Hz and 5.5 Hz data from Candy et al, 2001 that show facilitative and suppressive trends in the presence of a superimposed orthogonal mask are averaged). Also, the review does not seem to take into account data from extra-striate cortex for dichoptic masks (from Hou et al, 2020). More information about the exact data sets used from these studies could be listed in Table 1.
Furthermore, a consideration of the patterns of responses across the cortical surface in these studies, if available, so that they can be compared to the measurements across electrodes made in their study.

*As suggested, we now include information in Table 1 specifying, for each study, the figure from which the data were taken.*

*For the Candy et al data, we had no a priori reason to choose one frequency over the other, but wished to restrict the analysis to one data set per study (to avoid over-representing one study relative to the rest). We felt that averaging across frequencies was the fairest way to get an overall picture of the results from this paper. However we do agree with the reviewer that there are clear differences between those two frequencies, with 3.3Hz producing response gain and 5.5Hz contrast gain effects. We have therefore repeated the analysis using the data at each frequency (see below), and confirmed that this does not affect our group-level estimate of whether the posterior HDI of either parameter exceeds 1.*

3.3Hz	5.5Hz

*The reviewer makes an interesting suggestion about linking patterns of response in extra-striate regions and across the cortical surface to our changes in suppression across electrode location. But really the Hou et al data show a change from contrast gain in V1 to response gain in other areas, whereas our main observation is that contrast gain suppression increases at lateral electrodes. Furthermore, since source-imaged data are only readily available from a single study and a single mask type (dichoptic masks in the Hou et al. 2000 study), we could not see a principled way to include this systematically in the meta analysis. However we do now say in the Introduction:*

*“Work by Hou et al. (2020) used source-imaged EEG to study interocular suppression, and found evidence of a transition from contrast gain effects in V1 to response gain effects in extra-striate areas.”*

R1.2 Two-stage model
They use a two-stage model where the first stage fits a canonical gain control model to the unmasked data. They use a version of the model with 3 parameters to describe the contrast response function: a term that determines sensitivity (Z), another term that determines maximum response (Rmax) and a term (p) that determines whether the function accelerates or saturates. To incorporate the effect of the mask, they use a second-stage that modifies sensitivity and response gain. This model is used to analyze data from previous studies as well as their own data where they examine the effect of overlay, surround and dichoptic masks on the same targets.
Strengths: The modelling produces elegant summaries of previous data, despite the caveats noted above
Weakness: The model only considers the effect of the mask on contrast and response gain. However, their steady-state EEG data (first harmonic) show that the mask appears to have a significant effect on the acceleration parameter p, where the contrast response function goes from saturating without the mask to either accelerating (Fig 4b) or super-saturating (Fig 4 c, d, e) in the presence of the mask. Clearly the changes in saturation detract from a simple narrative centered on contrast and response gain, but it appears that this parameter is needed to better characterize the effect of the mask on the contrast response function.

*Contrast gain effects can appear to produce changes in acceleration/saturation, as can be seen by comparing the solid and dashed curves in Figure 1c. Essentially the effect of the mask is to shift the curve laterally, so that the accelerating portion of the curve occurs at higher contrasts, and the saturating part may not be apparent within the displayable contrast range. The model is able to produce these behaviours even with a fixed value of p (see e.g. the Hou et al. data in Figure 2). We have tried including a modulatory term on the p parameter when fitting the mask data. However this did not improve goodness of fit, and its distribution was centred around 1 for most data sets. It also had the effect of increasing the spread of the posterior for both of the gain parameters, making our estimates of them less precise. We therefore feel that this does not contribute much to the study, and have not included it in the manuscript. We do now say in the Methods section:*

*“Note that even with the value of p fixed by the first stage of modelling, the r and g parameters can accommodate apparent changes in the steepness of the contrast response function and the extent of saturation, by moving the steepest part of the curve to higher contrasts (g), or by reducing the available range of responses (r).”*

R1.3 The estimated model fits to the second harmonic term of the evoked response in the presence of a mask are particularly poor. Granted, the SNR of the second harmonic term is lower and the data are therefore noisier. It is not clear that the inclusion of the p term (acceleration) would improve the fit of the model, but a justification for not including the p term would be helpful. In general, it would be interesting to determine if including the change in the p parameter due to the mask, will change the reported effect of the contributions of contrast and response gains to the previous studies, and to their own first harmonic and second harmonic data.

*This is a consequence of several things, including the low SNR at the second harmonic as the reviewer points out. However, we think that much of the mismatch is due to the way the hierarchical modelling works. Essentially it is estimating parameter values for each data set (participant), and then working out an aggregate parameter value at the group level. The way that this aggregation operates does not necessarily produce the same behaviour as the averaged empirical data, hence the apparently poor fit in the group level data shown in Figure 6. We now report the R^2^ values in the figures, which are calculated across the individual participant data. See also R1.2 above for a discussion of allowing p to be a free parameter.*

R1.4 Minor point: Line 246. The authors mention that overall the first harmonic responses accelerate (median SI value 0.10). However, the preceding text says the SI values corresponding to acceleration are greater than 1

*Apologies for this error, it should be > 0 and < 0. We have now amended the text to talk about positive versus negative values.*

Reviewer #2: The authors characterized suppressive effects in SSVEP signals under conditions with different stimulus configurations. They concluded that cross-orientation suppression, interocular suppression and surround suppression can all be characterized in the form of contrast gain control for the first harmonics of SSVEP, but the second harmonics showed both contrast and response gain effects. The authors also explore changes across different electrode sites and different response frequencies and found that suppression generally became stronger at more lateral electrode sites, which suggests that suppression builds up across successive stages of processing. There have been many electrophysiological studies on the normalization properties in animal researches and human EEG. Although animal studies have reached conclusion on gain control effects for different stimulus properties, studies on EEG signals from human brain are not conclusive, as the authors pointed out in their meta-analysis on data from 16 published studies. The results based on newly collected data provided a more systematical pictures about gain control effects in SSVEP. The findings in this study is interesting and the paper is easy to follow. I generally have no serious concern with the work, but there are still a few comments or questions that the authors should address.

*We thank the reviewer for their supportive summary of our work, and for their insightful suggestions which we have responded to below.*

R2.1 The current title ‘Electrophysiological measures of visual suppression’ is a little overstated. ‘Electrophysiological’ includes studies with intracellular, extracellular recordings and EEG recordings. A more specific title might be better.

*Fair point. We have changed the title to: “Steady-state measures of visual suppression”.*

R2.2 Normalization Model and its fitting: a) there is no information about how well normalization model explains the meta-data and the data collected by new experiment. The authors should add this piece of information (the performance of the model on explaining the data) in the study (maybe in the paragraph between line 249 and 266). b) Many forms for gain control effects has been used, but only one specific form of gain control model was chosen in this study. Among different models, is the chosen one the best to explain the data? I understand that current study is modeling SSVEP signals, which might have a model different from models for spike activity, but at least there should be some discussions about why the chosen is good compared to other models for population response (VEP, the LFP and the MUA). The models for the normalization of the LFP in visual cortex in a recent paper by Wang et al. (2021, Superimposed gratings induce diverse response patterns of gamma oscillations in primary visual cortex) might be worth considering and discussing. c) It is clear for the procedures of model fitting with two steps, but I could find the reason why the authors fitting the data in this way. A few sentences to justify such fitting procedures might be good for readers. Is the two-step fitting similar to fitting a complete model to the data with one step?

*a) We have calculated the R^2^ values for each model fit, and now report these in Figures 2, 4 and 6. Note that this required us to rerun the fitting, and because of the stochastic nature of Markov Chain Monte Carlo the figures look very slightly different to our first draft.*

*b) Thank you for alerting us to the Wang paper, which we were not aware of. This is a very interesting study. The model presented in that paper (their equations 4, 8 & 9) is equivalent to our contrast gain change model for a fixed mask contrast (as here), albeit with different terminology (and with their b parameter from eq 10 set to 1 in our case). We have added a new section to the discussion (‘Model variations and other types of suppression’) where we compare different model variants and types of suppression.*

*c) We fitted the model in this way to ensure that the parameters used to fit the baseline data (p, Z and k) did not absorb any of the variance in the data with the mask present. This means that the estimates of the suppression parameters (r and g) are not affected by those other parameters being free. It also means that the same baseline model is used for each masking data set, which gives a fairer comparison across mask types than if the other three parameters were allowed to differ across mask types. We now say:*

*“Our rationale for fitting the baseline data first is that the values of the p, Z and R_max_ parameters are then fixed, and cannot trade off with the suppression-specific parameters (r and g) when fitting the masking data. This gives a cleaner estimate of the relative effects of each type of suppression.”*

R2.3 It is interesting to see different gain control effects between first and second harmonics: what is the cause for the difference? Some discussion about the difference or the origins of F1 and F2 will be good. Is there any possibility that the second harmonics is contaminated/affected by alpha rhythm around 10 Hz? In figure 3a, there seem to be dips around the two harmonic frequencies (5Hz and 10Hz), is this due to spectral analysis or normalization for power spectrum?

*Potentially the alpha rhythm might be responsible for the reduced SNR at the second harmonic. This was an oversight on our part – in more recent studies we have used a slightly higher fundamental (e.g. 7Hz) so that the second harmonic lies above the alpha band. However we do not think that alpha rhythms would otherwise affect our results, because we used a coherent averaging method (see R2.4) that substantially reduces noise at non-signal phase angles. We have added the following to the Discussion section:*

*“Second harmonic responses have previously been associated with different classes of neuron from the fundamental response (e.g. McKeefry et al., 1996), and imply the presence of one or more nonlinearities, such as squaring or rectification (Norcia et al., 2015; Regan & Regan, 1988). In principle a stronger nonlinearity might also result in the greater saturation we observe here at 10Hz.”*

*On the dips – yes, this is because the amplitude at the signal frequency is very strong, and so it results in SNRs slightly below 1 in the adjacent frequency bins. This is a common observation in SSVEP studies, but is not a concern because we do not use the SNRs at these frequencies for anything other than visualising the spectrum in Figure 3a.*

R2.4 Lines 186-187, ‘each condition were then coherently averaged (i.e. taking both the phase and amplitude into account)’. It is not clear what coherently averaged mean. Please clarify this procedure.

*Amended to:*

*“(i.e. averaging the full complex Fourier spectrum, including both phase and amplitude components)”*

R2.5 Lines 220-221, ‘However, the 95% highest density interval of the group posterior distribution for contrast gain control, shown in the final row, overlapped with…’ This is not a complete sentence.

*Thanks, now amended to ‘overlapped with a value of 1’.*

R2.6 Lines 280-282: There is no statistics report for the comparison between surround suppression and monocular/dichoptic suppression, and in line 292-285, the comparison between parietal regions and occipital regions.

*This is a really helpful suggestion. Because we already have the full posterior parameter distributions, we can make comparisons between mask conditions and electrode locations by calculating a p-value using the posteriors. These values have been inserted into the text as follows:*

*“We can use the posterior parameter distributions to calculate a Bayesian equivalent of a p-value for these comparisons to determine statistical significance. This analysis shows that at Oz less than 5% of the posterior distribution for aligned surrounds (p = 0.006) and orthogonal surrounds (p = 0.003) equals or exceeds the mean weight for monocular overlay masks (these are the distributions shown along the y-axes of Fig 4g, i and j). Suppressive weights also increase at bilateral electrodes over more parietal regions of cortex, particularly for surround suppression. For example, the weights are greater at electrode P7 than Oz for both aligned (p = 0.001) and orthogonal (p = 0.002) surrounds.”*

R2.7 Discussion ‘Contrast and response gain in other experimental paradigms’, Not all normalization can be characterized by contrast or response gain controls (see Wang et al. 2021 mentioned in comment 2C).

*Our reading of the Wang paper is that the effects they report are contrast gain effects, because the suppression between stimulus components occurs on the denominator of the gain control equation, and acts to shift the contrast response function to the right. However the reviewer is quite right that other forms of normalization, such as subtraction, are possible. We have included a new section in the Discussion headed ‘Model variations and other types of suppression’ to address these points.*
